# Retinal Outcomes in Diabetes: Antihyperglycemic Therapy, EWDR, and Perioperative Considerations

**DOI:** 10.3390/biomedicines14050963

**Published:** 2026-04-23

**Authors:** Tongyu Wang, Jiling Zeng, Mengquan Tan, Meiling Zhong, Huixian Zhou, Yaling Dai, Siyuan Song

**Affiliations:** 1 Shanghai Diabetes Institute, Shanghai Key Laboratory of Diabetes Mellitus, Shanghai Key Clinical Center for Metabolic Diseases, Shanghai Jiao Tong University Affiliated Sixth People’s Hospital, Shanghai 200233, China; 2 Department of Internal Medicine, Nazareth Hospital, Philadelphia, PA 19152, USA; 3 Institute of Rehabilitation Industry, Fujian University of Traditional Chinese Medicine, Fuzhou 350122, China; 4 Department of General Practice, The Third People’s Hospital of Shenzhen, Shenzhen 518114, China; 5 Department of Internal Medicine, Montefiore Medical Center, Albert Einstein College of Medicine, New York, NY 10461, USA; 6 Fujian Key Laboratory of Aptamers Technology, Fuzhou General Teaching Hospital (The 900th Hospital), Fujian University of Traditional Chinese Medicine, Fuzhou 350122, China; 7 Department of Anesthesiology, Montefiore Medical Center, Albert Einstein College of Medicine, Bronx, NY 10467, USA; 8 Department of Neuroscience, Baylor College of Medicine, Houston, TX 77030, USA

**Keywords:** diabetic retinopathy, retinal neurovascular unit, early worsening of diabetic retinopathy, HbA1c reduction, glycemic variability, GLP-1 receptor agonist, SGLT2 inhibitor, perioperative glycemic management, diabetic macular edema, VEGF

## Abstract

Diabetic retinopathy (DR) is a common cause of vision loss in diabetes, and it often progresses without early symptoms. DR reflects injury of the retinal neurovascular unit (NVU), which includes neurons, Müller glia, astrocytes, endothelial cells, pericytes, and immune cells. Chronic hyperglycemia drives oxidative stress, advanced glycation end products–receptor for advanced glycation end products (AGE–RAGE) signaling, mitochondrial injury, and low-grade inflammation. These changes disrupt endothelial junctions, promote leukostasis, weaken pericyte support, increase basement membrane thickening, and lead to capillary dropout and hypoxia. Hypoxia-related signaling increases anti-vascular endothelial growth factor (VEGF) activity, which raises vascular leakage and supports neovascular disease. Glial stress and microglial activation add cytokines and reactive oxygen species, and neural dysfunction can appear early and can weaken neurovascular coupling. Modern diabetes care changes the short-term risk landscape because potent therapies can lower HbA1c quickly. Large and rapid HbA1c reductions can trigger early worsening of diabetic retinopathy (EWDR), mainly in patients with high baseline HbA1c and moderate-to-severe baseline DR. Semaglutide’s retinopathy complication signal in SUSTAIN-6 fits an EWDR-like pattern that tracks with rapid glycemic improvement in vulnerable eyes. In parallel, surgery adds acute stress, inflammation, glucose swings, hemodynamic shifts, and medication interruptions. These factors can worsen microvascular instability during recovery. Current perioperative guidelines and regulatory recommendations describe glucose targets and medication safety considerations, including preoperative interruption of SGLT2 inhibitors to reduce euglycemic ketoacidosis risk; however, the retina-specific implications of these measures remain indirect. This review summarizes current evidence linking NVU biology, EWDR risk, and perioperative diabetes-related factors. It discusses how these factors may interact in patients with diabetes and how they may influence retinal outcomes. The review is intended to synthesize current evidence and mechanistic interpretations rather than to provide formal clinical practice recommendations.

## 1. Introduction

Diabetic retinopathy (DR) is a major cause of vision loss in people with diabetes [1,2]. The global burden of DR is likely to increase further because diabetes itself is rising worldwide. The IDF Diabetes Atlas estimated that 537 million adults aged 20–79 years were living with diabetes in 2021, and this number is projected to rise to 643 million by 2030 and 783 million by 2045 [2]. Recent global estimates also suggest that in 2020 DR caused about 1.07 million cases of blindness and about 3.28 million cases of moderate or severe vision impairment worldwide [3]. It often develops without symptoms in the early stage, so many patients are diagnosed only after retinal damage has already started [4]. With time, DR can progress from mild non-proliferative changes to severe disease with retinal ischemia, abnormal new vessel growth, vitreous hemorrhage, tractional retinal detachment, and permanent visual impairment [5]. Diabetic macular edema can occur at many stages and is one of the main causes of reduced central vision [6]. These clinical outcomes show that DR is not only a vascular disease. It is a multi-cell disorder that involves vascular injury, inflammation, and neural dysfunction in the retina [7,8].

DR has traditionally been described as a microvascular complication driven by chronic hyperglycemia [4]. Current models place the retinal neurovascular unit (NVU) at the center of disease initiation and progression [7,8]. The NVU includes retinal neurons, Müller glia and astrocytes, vascular endothelial cells, pericytes, and immune cells such as microglia and circulating leukocytes [8,9]. These cell types work together to match blood flow to neural activity, maintain the blood–retinal barrier, and control local energy balance [9,10]. These functions also act as endogenous retinal defenses against diabetes-related injury. In the early phase of diabetes, the retinal neurovascular unit can partly compensate through barrier maintenance, glial metabolic support, and bloodflow regulation. Clinically manifest DR therefore usually does not appear immediately after diabetes onset. Instead, it often emerges after a prolonged subclinical phase as compensatory reserve gradually declines and cumulative injury increases. Diabetes disrupts this system through oxidative stress, advanced glycation end products, mitochondrial injury, and persistent low-grade inflammation [11,12]. These processes injure endothelial cells and pericytes, weaken barrier function, and promote capillary dropout [7,11]. At the same time, glial cells and microglia shift toward inflammatory states and release cytokines and reactive oxygen species, which further damage vessels and neurons [8,12]. Retinal neurons can also show early functional changes, including altered synaptic signaling and impaired neurovascular coupling [13]. In many patients, these neural and inflammatory changes may appear before late vascular complications and may shape the pace of later progression [8,13]. This inflammatory component is not limited to a nonspecific low-grade state. It includes defined cytokines, adhesion molecules, inflammasome signaling, and immune-cell phenotype shifts that are now viewed as part of the core disease process in DR.

Recent clinical and imaging studies support this early-neurodysfunction framework. Multifocal electroretinography abnormalities have been reported in patients with diabetes even before clinical signs of DR are visible on routine examination. In parallel, reduced retinal neurovascular coupling has been linked with inner retinal thinning, especially ganglion cell layer thinning, in early DR. Together, these findings support the view that neuronal dysfunction and neurovascular uncoupling are not only late consequences of microvascular disease, but may also appear during earlier disease stages [14,15].

Systemic metabolic status affects the retina through several linked pathways. High glucose activates glycation reactions and stress signaling, increases oxidative injury, and disrupts vascular tone regulation [16,17]. Dyslipidemia can worsen endothelial dysfunction and inflammatory responses [18]. Hypertension increases mechanical stress on small vessels and raises the risk of leakage and nonperfusion [16]. Kidney disease often tracks with worse DR, which likely reflects shared microvascular injury and a higher inflammatory burden [19]. These systemic factors do not act alone. They interact and can change retinal perfusion, vascular permeability, and immune activation, which helps explain why DR risk depends on more than HbA1c alone [20].

Recent clinical practice has also changed the way systemic care and short-term physiology can shape retinal outcomes. Many modern glucose-lowering therapies can reduce HbA1c quickly, and large HbA1c drops can occur over a short period after treatment intensification [21]. Rapid glycemic improvement has been linked to early worsening of diabetic retinopathy (EWDR), which refers to a short time window when retinopathy lesions can worsen soon after glucose control improves [21,22]. This effect is most often reported in patients with poor baseline control and established moderate-to-severe retinopathy [21]. This creates an important interpretive point: long-term glycemic control protects the retina, but rapid glycemic improvement may transiently increase risk in vulnerable eyes during the early phase after treatment intensification [23].

Surgery is also common in people with diabetes, including ophthalmic procedures such as cataract surgery and vitreoretinal surgery, and many non-ophthalmic operations. The perioperative period often includes fasting, pain, stress hormone release, inflammation, and changes in hemodynamics [24]. These factors can cause acute hyperglycemia and large glucose swings. Blood pressure changes, blood loss, anemia, and fluid shifts can alter tissue perfusion, and the retina is sensitive to changes in oxygen delivery and microvascular flow [25,26]. Medication interruptions add another layer of risk. Some drugs are held before surgery for safety reasons, and this can increase hyperglycemia or ketone risk if insulin is not adjusted in a structured way [25]. Current perioperative guidelines and clinical reviews describe glucose targets and medication safety considerations, including preoperative interruption of SGLT2 inhibitors to reduce euglycemic ketoacidosis risk [27]. These perioperative issues are usually framed as general safety measures, but they may also influence retinal physiology through inflammation, endothelial stress, and altered perfusion [26].

This review summarizes current evidence on how modern antihyperglycemic therapies, EWDR, and perioperative factors may interact in shaping retinal outcomes in diabetes. Because the review includes clinical studies, preclinical findings, and mechanistic interpretation, its aim is to provide an evidence-based overview rather than formal guidance for clinical practice.

## 2. Pathobiology: Why Systemic Metabolism and Acute Stress Matter to the Retina

### 2.1. The Retinal Neurovascular Unit as the Disease Substrate

The retina needs a steady supply of oxygen and fuel because it is active all the time. This supply is managed by the retinal NVU [28]. The NVU includes vascular endothelial cells, pericytes, Müller glia, astrocytes, microglia, and retinal neurons such as ganglion cells, bipolar cells, and amacrine cells [28]. These cells work together to keep blood flow matched to neural demand, to keep the blood–retinal barrier intact, and to prevent fluid buildup in retinal tissue [28]. When diabetes disrupts this unit, vascular leakage, poor perfusion, inflammation, and neural dysfunction can develop at the same time [29].

An important point is that DR pathogenesis reflects not only progressive injury, but also gradual failure of endogenous retinal defense. In the early phase of diabetes, homeostatic functions of the NVU can buffer part of the metabolic and inflammatory stress. Over time, however, persistent hyperglycemia and related systemic stress weaken this protective capacity. Overt DR develops when cumulative injury exceeds this compensatory reserve.

Hyperglycemia injures endothelial cells through several connected pathways. Excess glucose increases reactive oxygen species (ROS) in mitochondria, and it activates NADPH oxidase, so oxidative stress rises [30,31,32]. High glucose also increases advanced glycation end products (AGEs), and AGEs bind to the Receptor for advanced glycation end products (RAGE) receptor on endothelial cells and immune cells, so NF-κB signaling increases [30,32,33]. This promotes inflammatory gene expression, including TNF-α, IL-1β, IL-6, and chemokines such as CCL2 [30,33]. Endothelial cells then increase adhesion molecules such as ICAM-1 and VCAM-1, so leukocytes adhere more easily to the vessel wall [33]. This leukostasis slows capillary flow, and it damages endothelial cells, and it increases local ischemia [33]. At the same time, protein kinase C (especially PKC-β) becomes more active, and this reduces nitric oxide signaling and increases endothelin-1 effects, so vascular tone becomes less stable [32]. Endothelial barrier proteins such as occludin and claudins can be reduced or disorganized, so permeability increases and the blood–retinal barrier becomes leaky [30,31,34].

Pericytes are key stabilizers of retinal capillaries. Diabetes can trigger pericyte apoptosis through oxidative stress and inflammatory signaling. Pericytes depend on PDGF-B/PDGFR-β signaling from endothelial cells for survival, and this support can weaken in diabetes [35]. Angiopoietin-2 can rise in stressed vessels, and this can destabilize the endothelium through the Tie2 pathway, and it can worsen leakage and inflammation [36]. As pericytes are lost, capillaries become fragile [35]. Microaneurysms form more easily, and focal leakage increases. Pericyte loss also changes capillary contractility, so local flow control becomes less precise.

Basement membrane thickening is another early structural change. Diabetes increases extracellular matrix production by endothelial cells and pericytes, including collagen IV, laminin, and fibronectin, and matrix turnover becomes abnormal [35]. This thickened basement membrane alters oxygen diffusion and cell–matrix signaling, and it can make capillaries less adaptable. Capillary dropout can then occur, so nonperfusion increases, and retinal hypoxia becomes more common [37,38]. Hypoxia stabilizes HIF-1α in retinal cells, and HIF-1α increases vascular endothelial growth factor (VEGF) expression. VEGF increases vascular permeability and promotes neovascular growth in advanced disease. VEGF also interacts with inflammatory signaling, so leakage and inflammation can amplify each other [39].

Glial cell populations are unlikely to contribute in the same way or at the same stage of DR. Müller cells may act as early stress responders because they directly regulate glutamate clearance, potassium balance, and water transport [40]. When these functions weaken, neuronal stress can rise and edema risk can increase [41,42]. This makes Müller cells especially relevant to early homeostatic failure and to fluid imbalance in the diabetic retina.

Microglia appear to contribute more strongly to inflammatory amplification. In diabetes, activated microglia release cytokines, complement-related signals, and inflammasome mediators that can worsen endothelial injury, barrier leak, and neuronal dysfunction [40,41,43]. This process is not a simple on–off switch. In current studies, retinal microglia more often show a shift toward a pro-inflammatory state, while compensatory anti-inflammatory or reparative responses appear insufficient to restore retinal homeostasis. Current evidence therefore supports a model in which Müller cells are more closely linked to early support failure, whereas microglia are more closely linked to inflammatory escalation and persistence.

Inflammation in DR involves a defined set of mediators rather than a nonspecific low-grade state. Clinical and translational studies have repeatedly linked DR to higher levels of TNF-α, IL-1β, IL-6, CCL2/MCP-1, ICAM-1, VCAM-1, and VEGF. At the pathway level, advanced glycation end products–receptor for advanced glycation end products (AGE–RAGE) and NF-κB signaling promote inflammatory gene expression, while NLRP3 inflammasome activity supports IL-1β release and inflammatory amplification. These pathways interact with VEGF signaling and help drive barrier leak, leukocyte adhesion, edema, and progressive microvascular instability [33,35,44,45,46].

Astrocytes are also part of the NVU and remain relevant to vascular support and neurovascular coupling [47]. However, compared with Müller cells and microglia, their temporal role in human DR is less clearly defined [40,48]. This difference matters because these glial populations are not interchangeable therapeutic targets [40,48]. Current translational work more often positions Müller cells in relation to edema and homeostatic failure, and microglia in relation to inflammatory control, while astrocyte-directed strategies remain less developed [40,49].

Neurodegeneration is increasingly viewed as an early part of DR rather than only a late consequence of overt microvascular disease [50]. Clinical electrophysiology, optical coherence tomography (OCT)-based structural studies, and neurovascular coupling studies all support early neural involvement [15]. At the same time, the exact order of events remains uncertain, because vascular and neural abnormalities often overlap and may progress together. Current evidence therefore supports early neural dysfunction, but it does not prove that neuronal injury always precedes microvascular change in every patient [51,52].

**Figure 1:**
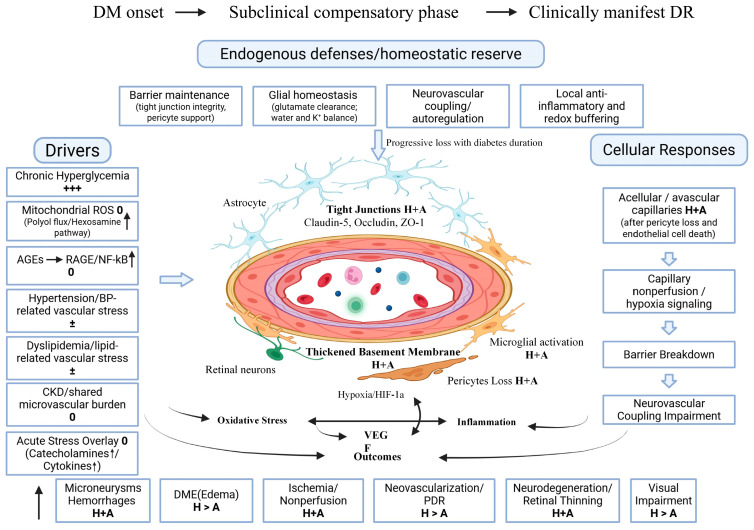
Progressive retinal neurovascular unit (NVU) injury in diabetes: endogenous defense failure, evidence-graded drivers, and human versus experimental correlates. This figure shows how diabetes affects the retinal NVU over time. Clinically manifest diabetic retinopathy (DR) is often delayed after diabetes onset and follows a prolonged subclinical compensatory phase. The upper panel highlights endogenous retinal defenses, including barrier maintenance, glial homeostasis, neurovascular coupling, and local redox/anti-inflammatory buffering. These protective functions gradually decline with increasing diabetes duration. On the left, the main drivers of NVU injury are shown. In the center, diabetes disrupts endothelial junctions, thickens the basement membrane, causes pericyte loss, activates microglia, and stresses retinal neurons and glia. On the right, these changes progress to acellular/avascular capillaries, capillary nonperfusion with hypoxia signaling and increased vascular endothelial growth factor (VEGF), barrier breakdown, and impaired neurovascular coupling. At the bottom, the main tissue and clinical outcomes include microaneurysms and hemorrhages, diabetic macular edema, ischemia/nonperfusion, neovascularization, neurodegeneration/retinal thinning, and visual impairment. Notation: +++, supported by multiple clinical trials; ±, supported by some, but not all, clinical trials; 0, no direct clinical-trial evidence in DR; H, documented in humans; A, reproduced in experimental diabetic animals; H + A, supported in both; H > A, prominent in humans but incompletely reproduced in common diabetic rodent models.

Neurovascular coupling depends on signaling among neurons, glia, endothelial cells, and pericytes [28]. Neuronal activity normally triggers glial calcium signals, and this leads to release of vasoactive mediators such as nitric oxide and prostaglandin-related signals, so vessels dilate in active regions. In diabetes, nitric oxide availability can fall, endothelin-1 signaling can rise, and pericyte function can become abnormal. Blood flow responses then become weaker or delayed, so oxygen delivery does not match demand, and local hypoxia stress becomes more likely even without large vessel disease [28,52] (Figure 1). This delayed transition from diabetes onset to clinically manifest DR is consistent with duration-based clinical data showing that retinopathy prevalence remains low within the first 10 years of type 2 diabetes and increases progressively thereafter [53].

Systemic nutritional and inflammatory indices may also reflect part of the inflammatory burden linked with DR. Recent studies have reported that lower prognostic nutritional index (PNI), which combines serum albumin and lymphocyte count, is associated with DR. Other blood-based inflammatory indices, such as the neutrophil-to-lymphocyte ratio and the systemic immune-inflammation index, have also been linked with DR or diabetic microvascular complications in observational studies. These markers are best viewed as clinical correlates of systemic inflammatory stress rather than established retina-specific causal mechanisms [54].

Taken together, current evidence suggests that early DR involves overlapping neural, glial, and microvascular stress [14,55]. Müller cells may be especially relevant to early homeostatic failure, microglia to inflammatory amplification, and neurovascular dysfunction to early functional decline [48,55,56]. However, the exact temporal sequence and relative weight of these processes in human DR remain incompletely defined [14,55].

### 2.2. Glycemic Variability vs. Mean Glycemia

HbA1c reflects average glycemia over about two to three months. It does not capture daily peaks, rapid drops, and time spent in extreme glucose ranges [57,58]. Two patients with the same HbA1c can have very different glucose patterns. One patient can have stable glucose values, and another patient can have large swings. These swings can matter to the retina because endothelial cells and immune cells respond strongly to acute stress signals [59,60].

For diabetic retinopathy, the relevant glucose changes are not simply the brief rises that follow meals. Postprandial blood glucose elevation is common and usually short in duration, but it is not the main pattern emphasized in the DR literature discussed here. In contrast, the changes that appear more relevant to DR are larger and more sustained. These include repeated wide glucose swings, prolonged exposure to marked hyperglycemia, and rapid downward shifts in glycemia that occur over weeks to months during treatment intensification [57,58,59,60]. This distinction is important because short postprandial excursions and broader glycemic instability do not reflect the same biological stress on the retina.

Larger and more sustained glucose changes can increase oxidative stress and inflammatory signaling more strongly than brief physiologic excursions. Rapid glucose rises can increase ROS generation and activate PKC and AGE–RAGE signaling. Rapid glucose drops can change osmotic balance across the retinal vasculature and glia. Water movement can then shift quickly, which may affect tissue hydration and edema tendency, especially when barrier function is already weak [59,60,61]. Large swings can also trigger counter-regulatory hormones such as epinephrine and cortisol, and these hormones can increase hepatic glucose output and worsen inflammation-related signaling [59,60]. In this setting, glycemic variability may further increase oxidative and inflammatory stress in the NVU [59,60,61].

It is hard to prove that glycemic variability alone causes DR progression because variability is linked with other risk factors, such as longer diabetes duration, insulin use, kidney disease, and advanced baseline DR [62,63,64]. Even so, clinical reports repeatedly show that large and fast HbA1c reductions are linked with short-term retinopathy worsening [21]. This supports the idea that the size and speed of metabolic change can stress the NVU during the early phase of treatment intensification [21].

### 2.3. EWDR as a Systems-Level Phenomenon

EWDR describes a short period when retinopathy activity increases soon after rapid improvement in glycemic control. Clinical reports and reviews consistently show that this pattern is most often seen in patients with high baseline HbA1c and moderate-to-severe baseline DR, and that it usually appears within the first weeks to months after treatment intensification [21,65,66].

The biological basis of this pattern is still not fully defined, but several mechanisms have been proposed. One proposed explanation is that rapid glycemic correction may disturb retinal bloodflow regulation in eyes that already have capillary nonperfusion and limited vascular reserve [66,67]. In this setting, altered perfusion may increase local hypoxia signaling and may contribute to VEGF-related leakage, especially when baseline ischemic stress is already present [66,67]. This mechanism is biologically plausible and is supported by indirect clinical inference, but direct human evidence remains limited.

A second possible explanation involves osmotic and inflammatory change. Rapid shifts in glycemia may alter plasma osmolarity and transvascular fluid movement. In eyes with a weakened blood–retinal barrier, this may contribute to retinal thickening or macular edema. At the same time, oxidative and inflammatory signaling may also change during this transition, which could further affect endothelial junction stability [49,65,66,68]. This explanation is also supported mainly by biologic rationale and indirect evidence rather than by direct demonstration in all human EWDR cases.

These pathways are not mutually exclusive, and direct human evidence does not show that every pathway operates in every case of EWDR. Instead, current clinical evidence supports EWDR as an observed early worsening pattern, while current mechanistic evidence supports a multifactorial explanation rather than a single uniform pathway [21,65,66,67].

## 3. Long-Term Glycemic Control: The “Metabolic Memory” Foundation for Retinal Protection

Strong evidence shows that long-term glycemic control lowers the risk of DR and slows its progression [69,70,71,72,73]. This conclusion comes from large randomized trials and long follow-up studies. These studies show two consistent patterns. Better glycemic control reduces microvascular injury in the retina over years. Retinal benefit can also persist even when HbA1c differences between treatment groups become smaller later [70,71,72,73]. This persistent effect is often called “metabolic memory” or the “legacy effect.” It suggests that early exposure to lower glucose levels leads to long-lasting protection of retinal tissue [68,70,74].

The concept of metabolic memory fits with what is known about how chronic hyperglycemia damages the retinal neurovascular unit. Endothelial cells, pericytes, Müller glia, microglia, and retinal neurons do not simply respond to glucose on a day-to-day basis. Instead, long-term hyperglycemia causes durable molecular changes that can persist after glucose improves [68,74,75,76]. One mechanism is the accumulation of AGEs in extracellular matrix and basement membranes. AGEs bind to RAGE and can keep NF-κB signaling active, which supports a sustained inflammatory state [68,74]. Another mechanism is persistent oxidative stress. Hyperglycemia increases mitochondrial ROS production and activates NADPH oxidases. This can lead to chronic damage to DNA, proteins, and lipids. It can also maintain activation of stress pathways such as PKC signaling [68,73,75,76]. Endothelial cells can then remain dysfunctional, with reduced nitric oxide signaling and increased endothelin activity, even after glucose improves [74,76]. Epigenetic changes also provide a plausible basis for metabolic memory. Hyperglycemia can alter histone modifications and DNA methylation in vascular and immune cells. These changes can maintain higher expression of inflammatory genes, adhesion molecules such as ICAM-1 and VCAM-1, and pro-leakage factors [77,78]. In the retina, these durable changes can keep the blood–retinal barrier fragile and can support leukocyte adhesion, microvascular occlusion, and chronic low-grade inflammation.

Long-term glycemic control can interrupt these processes. When average glucose is lower over years, AGE formation slows, oxidative stress is reduced, and inflammatory signaling pressure decreases [68,70,71,72,79]. Endothelial junction integrity is easier to maintain, and pericyte survival may be improved. Capillary closure and nonperfusion can also progress more slowly [70,71,72,73]. In parallel, lower glucose reduces glial stress. Müller glia are less likely to shift toward an inflammatory and dysfunctional state, and microglia activation pressure may decrease. Neural injury may also slow because the retinal environment becomes less inflammatory and less hypoxic [68]. These cellular effects provide a biologically plausible explanation for why early and sustained glycemic control can create long-term retinal benefit.

Clinical trial evidence supports this framework. In type 2 diabetes, large trials that used intensive glucose control reported lower rates of microvascular complications during the intervention, and later follow-up showed that some microvascular benefits persisted even when HbA1c differences between groups narrowed [69,70,71]. This pattern supports the idea that earlier glycemic exposure influences later tissue outcomes. Importantly, the retina is one of the microvascular beds where this long-term protection is clinically visible. Reduced risk of retinopathy progression often takes time to emerge, which matches the slow biology of capillary loss, basement membrane remodeling, and chronic inflammatory signaling [68,69,70].

Evidence from action to Control Cardiovascular Risk in Diabetes (ACCORD) Eye provides additional detail for patients with longer diabetes duration and high cardiovascular risk. In ACCORD Eye, intensive glycemic control reduced the risk of DR progression compared with standard control. The study also tested lipid therapy strategies, and fenofibrate added to statin therapy reduced DR progression compared with statin alone [71,80,81,82]. These results are important because they show that retinal risk is shaped by more than glycemia. Lipid-related pathways can influence endothelial inflammation and vascular permeability. Fenofibrate activates PPAR-α signaling, which can reduce triglycerides and can also affect inflammatory gene expression. In vascular cells, PPAR-α activation can reduce NF-κB-driven inflammation and can improve endothelial function. These actions provide a plausible link between systemic lipid therapy and slower retinopathy progression. The ACCORD Eye data therefore supports a broader view of retinal protection. It suggests that strong retinal benefit comes from coordinated control of glucose and systemic vascular risk factors, not from glucose alone [80,81,82].

These trial findings also explain a key clinical tension in modern diabetes care. Long-term glycemic improvement protects the retina. At the same time, large and rapid HbA1c reductions can increase short-term risk of early worsening of DR in vulnerable patients [61]. This short-term risk does not cancel the long-term benefit. This short-term risk does not negate the long-term benefit. Instead, it highlights the need to interpret early retinal events in the context of baseline retinal status and the magnitude of glycemic change [69,70,71,72,73,80,81].

### 3.1. Evidence Base

EWDR describes a short period of increased retinopathy activity that appears soon after rapid improvement in glycemic control [83,84]. This pattern has been reported in both type 1 and type 2 diabetes. It has been observed after intensive insulin therapy, after major treatment escalation in poorly controlled diabetes, and in settings where HbA1c drops quickly over a short time [65,83,84,85]. Systematic reviews consistently note the same key association. EWDR is most strongly linked to the size and speed of HbA1c reduction [65,83,85]. The worsening usually occurs early after treatment intensification, often within weeks to a few months [65]. This worsening is usually followed by longer-term benefits when good glycemic control is maintained [84].

Quantitative estimates remain heterogeneous across studies because definitions, retinal assessment methods, and follow-up windows differ. In a 2021 systematic review of 19 studies including 15,588 participants, reported EWDR prevalence ranged from 3.3% to 47% after intensification of glycemic control, with events reported from 3 to 84 months after treatment change. This wide range reflects major methodological differences rather than a single stable incidence estimate. The same review also showed that no universal threshold for “rapid” HbA1c reduction is accepted across studies. Some studies and reviews have used a fall of ≥1.0 percentage point over about 6 months as a marker of intensive glycemic improvement, whereas more recent real-world work in type 2 diabetes has defined “rapid” reduction as >1.5% within 12 months and “very rapid” reduction as >2.0% within 6 months. These definitions should be viewed as study-specific operational cutoffs rather than validated biologic thresholds [22,86].

The time course also differs across settings. In the DCCT, early worsening at 6 or 12 months was more frequent with intensive than conventional treatment, but many cases had improved by 18 months and long-term retinal outcomes still favored intensive control. This supports the view that EWDR is often a transient phenomenon, although not every early worsening event is clinically trivial and risk is higher when baseline retinopathy is more advanced [83,86].

Clinical descriptions from earlier intensive therapy eras provided detailed phenotype features. Patients could develop an increase in retinal hemorrhages, cotton wool spots, venous beading, intraretinal microvascular abnormalities, and progression toward proliferative changes [83]. Diabetic macular edema could also worsen in susceptible eyes. These changes were most often seen in patients who had long-standing poor control before intensification. This older literature remains important because it shows that EWDR is not restricted to one drug class. It is a general response pattern that can occur when the metabolic environment changes quickly in a retina that is already vulnerable [85].

**Figure 2:**
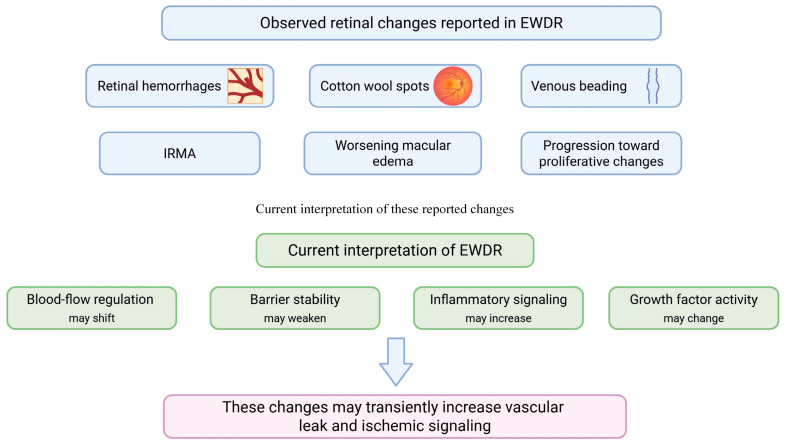
Clinical features and current interpretation of early worsening of diabetic retinopathy (EWDR) after rapid glycemic improvement. This figure summarizes the retinal changes reported in earlier descriptions of EWDR, including retinal hemorrhages, cotton wool spots, venous beading, intraretinal microvascular abnormalities, worsening macular edema, and progression toward proliferative disease. It also summarizes the current interpretation that EWDR reflects a transient stress response of the retinal neurovascular unit rather than a direct toxic effect of glucose lowering. In this model, retinal bloodflow regulation, endothelial barrier stability, inflammatory signaling, and growth factor activity may shift and may temporarily increase vascular leak and ischemic signaling.

The most widely accepted interpretation is that EWDR is not a direct toxic effect of glucose lowering. It reflects the stress response of the retinal neurovascular unit during rapid metabolic correction. During this period, retinal blood flow regulation, endothelial barrier stability, inflammatory signaling, and growth factor activity can shift. These changes can temporarily increase leakage and ischemic signaling, even when long-term glycemia is improving [28,65] (Figure 2).

EWDR should be viewed in a broader comparative context rather than mainly as a question of incretin-based therapy. The classic setting is intensive insulin treatment, especially in patients with poor baseline control and pre-existing retinopathy. Metabolic bariatric surgery is another important context because it can also produce large and rapid glycemic improvement over a short period. These comparisons matter because they support the view that EWDR is linked more closely to the speed and magnitude of metabolic change than to a single drug class [65,83,87].

### 3.2. Who Is at Highest Risk?

EWDR risk is not equal across all patients. It concentrates in a subgroup with advanced baseline retinal disease and severe baseline metabolic stress. The strongest clinical risk factor is the presence of moderate-to-severe baseline DR, especially severe non-proliferative disease or proliferative disease. In these eyes, the capillary bed is often already compromised, pericyte loss is more advanced, and the blood–retinal barrier is more fragile. Small physiologic shifts can lead to visible lesion progression because the system has limited reserve [21,65].

High baseline HbA1c and long-standing poor glycemic control also increase risk [21,57,63]. These features usually indicate sustained oxidative stress, accumulation of advanced glycation end products, persistent inflammatory activation, and capillary instability. In this setting, large HbA1c drops can occur soon after therapy intensification. The magnitude of HbA1c decline over a short time is repeatedly linked to EWDR risk [21,65]. This relationship supports the idea that the retina is sensitive to fast metabolic shifts, especially when baseline vascular and glial stress is already high [57,63].

Hypertension and kidney disease often track with EWDR risk [88,89,90]. These comorbidities reflect broader microvascular vulnerability. Hypertension increases mechanical stress on small retinal vessels and can worsen leakage and hemorrhage risk. Kidney disease correlates with systemic endothelial dysfunction, inflammation, and impaired vascular repair capacity [88]. When these systemic factors coexist with advanced DR, the retina may be more sensitive to acute changes in perfusion and inflammatory load during glycemic correction [89,90].

The risk setting is often a patient who has had uncontrolled diabetes for years and then starts a highly effective therapy. This includes intensive insulin therapy and potent incretin-based therapies. The common factor is not the drug class itself. The common factor is the rapid shift from a high-glucose state to a much lower-glucose state in a short time [57,63] (Table 1) (Figure 2).

Risk stratification should also distinguish between diabetes type and baseline retinal severity more clearly. Much of the classic EWDR literature came from type 1 diabetes and intensive insulin therapy, especially in patients with pre-existing mild to moderate retinopathy. By contrast, contemporary type 2 diabetes data are more mixed. For example, a large real-world study in type 2 diabetes with only mild or moderate non-proliferative diabetic retinopathy (NPDR) did not find an association between HbA1c reduction and retinopathy worsening, even when “rapid” and “very rapid” HbA1c reduction thresholds were applied. These findings suggest that EWDR risk is not uniform across all treatment intensification settings and is more likely to concentrate in patients with poorer baseline control and more advanced baseline retinal disease [22,83].

### 3.3. Biological Basis and Mechanistic Interpretation

EWDR can be described using linked cellular pathways in the retina. Rapid glycemic improvement can change retinal blood flow and autoregulation [91,92]. Endothelial nitric oxide signaling and endothelin signaling control vascular tone [92,93]. In diabetes, nitric oxide bioavailability is often reduced by oxidative stress, and endothelin activity can be increased [92,94]. When glycemia changes quickly, vascular tone regulation can shift [91]. Pericytes also contribute to capillary diameter control [95]. If pericytes are reduced, capillary tone becomes less stable [95]. This combination can change how perfusion is distributed across already damaged capillary beds [91,95]. Regions near the edge of nonperfusion may become more hypoxic or more variable in oxygen delivery [96].

Retinal hypoxia activates HIF-1α signaling [96]. HIF-1α increases transcription of VEGF and other hypoxia-related genes [33,96]. VEGF increases vascular permeability by affecting endothelial junction proteins and promoting vesicle transport [33,49]. In a retina with pre-existing ischemia, even small changes in perfusion pressure or capillary flow can change local hypoxia signaling. This can increase VEGF-driven leakage and can increase edema risk, especially in the macula [33,96].

Rapid metabolic change can also shift osmotic gradients. Glucose contributes to plasma osmolarity. A fast reduction in glucose can change fluid balance, and this can affect transvascular water movement when the blood–retinal barrier is already leaky [49]. Müller glia regulate water transport through aquaporin-4 and control extracellular potassium through Kir4.1. When Müller function is stressed, water handling becomes less stable. A fast osmotic change can then contribute to retinal swelling and can worsen macular edema.

Inflammation is another key part of this period. Hyperglycemia drives AGE–RAGE signaling, oxidative stress, and NF-κB activation [57]. When glycemia improves quickly, some stress signals decline while other responses shift [57]. Microglia can change activation state, and Müller glia can change cytokine release [36,57]. Endothelial cells can also change expression of adhesion molecules such as ICAM-1 and VCAM-1 [57]. Leukocyte adhesion and capillary plugging can worsen nonperfusion in some settings [57]. Inflammasome pathways such as NLRP3 may also contribute to cytokine release, including IL-1β, which can impair barrier stability [57]. These inflammatory transitions can support a short phase of lesion progression in susceptible eyes.

Growth factor pathways beyond VEGF can also play a role. Angiopoietin-2 can rise in stressed retinal vessels, and Tie2 signaling can fall, which destabilizes the endothelium and increases leakage [36]. PDGF-B/PDGFR-β signaling supports pericyte survival. If this support is impaired, pericyte loss and capillary fragility can worsen [36,95]. These pathways can interact with VEGF signaling and can amplify leakage and ischemic responses during the early phase after treatment intensification [33,36].

### 3.4. Interpretive Considerations Arising from Current Evidence

Published studies and reviews suggest that baseline retinal status and the tempo of glycemic change may influence short-term EWDR risk during treatment intensification. Several reviews have proposed that baseline retinal assessment may help contextualize short-term retinal risk in patients expected to undergo rapid HbA1c reduction. A dilated retinal exam provides staging information on non-proliferative and proliferative changes [97,98]. Optical coherence tomography can identify center-involving macular edema and can provide a baseline for follow-up [99]. OCT angiography can show capillary nonperfusion and may help define ischemic burden in selected cases [100]. This baseline assessment may be especially relevant when baseline HbA1c is very high, when the patient has long-standing diabetes, or when there is prior evidence of DR [65,98].

Long-term glycemic improvement remains important because its long-term benefit is strong [84]. At the same time, an unnecessarily abrupt HbA1c drop in patients with severe baseline DR may increase short-term risk, especially when a slower approach is clinically feasible. Staged therapy changes, careful insulin titration, and coordination with ophthalmology have therefore been discussed in reviews as possible ways to reduce this concern in high-risk eyes [65,98]. The specific pace of HbA1c reduction has not been defined as a single safe threshold for all patients, but clinical experience and reviews support the general principle that very large and very fast reductions carry higher EWDR risk in advanced DR [65].

The literature suggests that EWDR events are most often reported within the first weeks to months after treatment intensification. This pattern has led some authors to discuss whether closer early follow-up may be useful in selected high-risk settings, although formal evidence-based surveillance thresholds remain limited. EWDR events tend to occur within weeks to months [65], which suggests that early follow-up eye assessment may be more informative than routine annual screening in some high-risk settings [98]. Relevant findings may include new hemorrhages, progression of non-proliferative changes, new signs of neovascularization, and changes in macular thickness on OCT [99]. Visual symptoms such as new floaters, blurred central vision, or sudden vision changes may also be relevant during this period.

Retinal treatment remains important when concurrent retinal disease is present. In proliferative DR, prompt treatment reduces the risk of vitreous hemorrhage and tractional retinal detachment [101]. Anti-VEGF therapy can reduce neovascular activity and can also reduce macular edema [101,102]. Panretinal photocoagulation remains an established option for proliferative disease [101]. For center-involving diabetic macular edema, anti-VEGF is the standard first-line therapy in many settings, and focal or grid laser may be used in selected cases [102]. Taken together, these measures may help stabilize the retina during the systemic transition period while long-term glycemic benefit is maintained [84].

Perioperative periods may deserve particular attention when they overlap with therapy intensification. Surgery can increase stress hormones and inflammation, and it can cause glucose swings and hemodynamic changes. In a patient already at risk for EWDR, these factors may add to retinal vulnerability. Reviews have therefore discussed perioperative glucose stability, avoidance of prolonged hypotension and anemia, and careful coordination of medication changes as factors that may help limit overlapping physiological stress during this period [98].

## 4. Modern Antihyperglycemic Therapies and Retinal Outcomes

### 4.1. GLP-1 Receptor Agonists (GLP-1RAs) and Dual Incretin Therapies

#### 4.1.1. Evidence Signal: Semaglutide and Diabetic Retinopathy Complications

The clearest retinopathy safety signal in the modern drug era came from semaglutide in SUSTAIN-6, where diabetic retinopathy complications occurred in 3.0% of semaglutide-treated patients and 1.8% of placebo-treated patients [102,103]. The events were mostly early and were concentrated in patients with pre-existing DR, higher baseline HbA1c, and insulin use [103]. Post hoc analysis suggested that much of this effect could be explained by the magnitude and rapidity of HbA1c reduction during the first 16 weeks [103]. However, this explanation should not be presented as definitive. SUSTAIN-6 was not designed as a dedicated retinal imaging trial, baseline DR severity was not formally graded in a standardized way, fundus photographs were not centrally graded, and the retinopathy endpoint combined treatments and clinical events rather than stepwise grading of retinal change [103]. In addition, larger GLP-1RA datasets outside SUSTAIN-6 have not shown a uniform retinopathy signal, and recent systematic reviews remain inconclusive overall [104,105]. These points support an EWDR-like interpretation, but they also mean that drug-specific retinal effects, endpoint structure, and trial design factors cannot be fully excluded.

This “EWDR-like” framing also matches a meta-regression across GLP-1RA cardiovascular outcomes trials, where the magnitude of HbA1c reduction correlated with retinopathy risk [106,107,108]. This supports a simple clinical model. Retinopathy risk during the first months after therapy intensification tracks with the speed and size of glycemic change, especially when baseline HbA1c is very high and baseline retinopathy is already moderate to severe [22,107].

At the tissue level, the EWDR-like model is biologically plausible because rapid glycemic correction can change retinal hemodynamics and barrier stability in a short time [21,22]. Retinal endothelial cells and pericytes in diabetes already show oxidative stress, lower nitric oxide signaling, higher endothelin tone, and weaker tight-junction organization. In that setting, a fast systemic metabolic shift can change perfusion patterns in partially damaged capillary beds, and it can shift local hypoxia signaling. Hypoxia-responsive pathways, including HIF-1α-linked programs, can increase VEGF signaling in ischemic retina, and VEGF can further loosen endothelial junctions and raise leakage. Müller glia and microglia can add to this early-phase instability because they regulate water handling, glutamate homeostasis, and inflammatory tone. If glial stress responses increase during the transition period, cytokines such as IL-1β and TNF-α can further weaken barrier function and can worsen edema risk [21]. In this framework, semaglutide is best viewed as a potent trigger for rapid HbA1c reduction in high-risk patients, rather than as a retina-specific toxin [106,108].

#### 4.1.2. Broader GLP-1RA Evidence

Across the GLP-1RA class, retinopathy outcomes are more mixed than the SUSTAIN-6 signal might suggest. Many trials were not designed with detailed eye endpoints, and event definitions often relied on adjudicated complications or treatment events instead of standardized retinal imaging [104]. In several pooled analyses, most GLP-1RAs do not show a consistent statistically significant harm signal, and heterogeneity across trials is common. Across published trials, absolute retinopathy event risk appears low in patients without baseline DR, whereas interpretive caution may be more relevant in patients with moderate-to-severe baseline DR and very high baseline HbA1c, especially when large early HbA1c reductions are expected [109].

#### 4.1.3. Possible Protective Mechanisms over Longer Time Scales

Mechanistic work has proposed that GLP-1 signaling could support retinal resilience over longer durations. GLP-1 receptors have been reported in multiple retinal cell populations in experimental settings, and GLP-1 pathway activation is often linked to higher cyclic adenosine monophosphate (cAMP) and PKA signaling, with downstream effects that can reduce inflammatory gene expression and oxidative stress signaling in stressed tissues. In preclinical discussions, these effects are often framed as reduced microglial inflammatory activation, improved neuronal stress tolerance, and improved endothelial function [110,111]. These proposed mechanisms may help interpret long-term retinal protection, but they are not routine patient-level trial endpoints.

#### 4.1.4. Interpretation of Current GLP-1RA Evidence in Patients with Pre-Existing Retinal Disease

In patients with pre-existing retinal disease, the available literature raises the possibility that the early phase after GLP-1RA or dual incretin initiation may deserve closer interpretation, especially when rapid HbA1c reduction is expected. Baseline retinal phenotype appears relevant because EWDR-like patterns have been reported mainly in patients with pre-existing DR and large early glycemic shifts. However, this inference is based mainly on post hoc analyses and broader EWDR literature rather than on prospective retinal management trials. These observations are therefore best viewed as interpretive context rather than as formal practice recommendations [112].

### 4.2. SGLT2 Inhibitors (SGLT2i): Retinal Outcomes and Perioperative Implications

#### 4.2.1. Retinal Outcomes: Neutral-to-Beneficial Patterns in Observational Data, with Inconsistency

Randomized trials of SGLT2 inhibitors were not built around ophthalmic endpoints, so they provide limited direct information on diabetic retinopathy progression [113,114]. Real-world cohorts and meta-analyses have reported neutral-to-beneficial associations in some settings, including lower risk of sight-threatening retinopathy compared with several alternative drug classes in large observational comparisons [115,116]. At the same time, not all studies show benefit, and some analyses report no clear reduction in vision-threatening retinopathy [113,114]. This mix is expected because observational designs can be affected by confounding by indication, differences in healthcare use, and differences in baseline eye screening [115].

If a protective association is real, it could be driven by systemic effects that matter to the retinal neurovascular unit. SGLT2 inhibitors lower glucose with limited hypoglycemia, and they often lower blood pressure and reduce volume overload, and these changes can reduce vascular shear stress and endothelial strain [116]. Improved renal outcomes can also reduce systemic inflammation and endothelial dysfunction, and this could indirectly support retinal microvascular stability [113]. Some mechanistic discussions also propose anti-inflammatory effects, including lower oxidative stress signaling and lower innate immune activation in vascular beds, but the strength of retina-specific evidence varies and should be described as proposed rather than proven [114,115].

#### 4.2.2. Perioperative Safety: Euglycemic DKA Risk and Stopping Rules

The perioperative issue for SGLT2 inhibitors is clear and has direct safety consequences. Multiple authorities advise stopping SGLT2 inhibitors before scheduled surgery to reduce postoperative ketoacidosis risk, including euglycemic diabetic ketoacidosis (DKA). The U.S. Food and Drug Administration labeling updates advise stopping canagliflozin, dapagliflozin, and empagliflozin at least 3 days before scheduled surgery, and stopping ertugliflozin at least 4 days before.

These recommendations are relevant to the present review because perioperative ketoacidosis, dehydration, and hemodynamic instability may increase systemic stress and could, in theory, aggravate retinal vulnerability [117]. However, direct retina-specific evidence in this setting remains limited.

### 4.3. Insulin Intensification

Insulin remains the classic setting for EWDR and provides the strongest historical comparison for newer therapies. In the DCCT, early worsening at 6 or 12 months was observed in 13.1% of intensively treated patients and 7.6% of conventionally treated patients. About half of these early changes had improved by 18 months, and long-term retinal outcomes still favored intensive treatment. These results support two points. First, rapid glucose improvement can increase short-term retinal risk in susceptible eyes. Second, this short-term risk does not cancel the long-term retinal benefit of stronger glycemic control. This historical context is important because it shows that EWDR is not a new phenomenon created by GLP-1RAs or other modern drug classes [83,118].

This historical comparison is also useful when interpreting newer therapies. It suggests that EWDR is better understood as a rapid-glycemic-improvement phenomenon than as a drug-class-specific effect. This is why insulin intensification remains an essential reference point when considering EWDR signals reported with GLP-1 receptor agonists or other modern therapies.

### 4.4. Metabolic Bariatric Surgery as a Rapid-Glycemic-Improvement Setting

Metabolic bariatric surgery is another important comparative setting for EWDR because it can produce abrupt and large improvements in glycemia. This makes it useful for interpretation because the exposure pattern is similar in principle to other rapid glucose-lowering settings, even though the intervention is surgical rather than pharmacologic. A systematic review and meta-analysis found that bariatric surgery was associated with lower long-term prevalence of all DR and sight-threatening DR compared with medical management. At the same time, early worsening can occur, especially in patients with more severe baseline DR. These findings again support a two-phase interpretation: rapid metabolic improvement may increase short-term retinal instability in vulnerable eyes, while long-term retinal burden may still decrease over time. This comparison helps place GLP-1RA-associated signals into a broader framework and reduces the risk of overattributing EWDR to any single drug class [87].

### 4.5. Metformin

Metformin is generally viewed as neutral-to-beneficial for microvascular risk because it improves glycemia and insulin sensitivity and reduces weight gain risk compared with several alternatives [119,120]. Mechanistic discussions often focus on AMP-activated protein kinase (AMPK)-linked effects that can reduce hepatic glucose output and can alter inflammatory and oxidative stress signaling in some tissues [120], but direct clinical evidence that metformin changes diabetic retinopathy progression independent of glycemic change is limited [119]. In perioperative care, the main concerns relate to lactic acidosis risk in severe renal dysfunction or hemodynamic instability, so perioperative decisions are usually driven by systemic safety rather than by retina-specific endpoints [119,120].

### 4.6. DPP-4 Inhibitors, Sulfonylureas, and Thiazolidinediones

DPP-4 inhibitors usually produce modest HbA1c reductions, so they are not a typical trigger for EWDR through rapid glycemic shifts [121,122]. Observational evidence on retinopathy risk has been mixed, and at least one recent meta-analysis did not find a clear association between DPP-4 inhibitor use and diabetic retinopathy risk overall [121,122,123].

Sulfonylureas are more relevant to perioperative and short-term safety because they increase hypoglycemia risk, and hypoglycemia can increase glycemic variability. Large swings in glucose can complicate perioperative management and can increase systemic stress responses, so their indirect relevance to retinal vulnerability is mainly through safety and variability rather than a specific retinopathy signal [124].

Thiazolidinediones such as pioglitazone activate PPARγ and can cause fluid retention. Case reports and safety discussions have raised concern that fluid retention may worsen diabetic macular edema in susceptible patients, although population-level evidence has not always shown a strong association. In a patient with existing macular edema or with high edema risk, closer monitoring after thiazolidinedione (TZD) initiation is a reasonable precaution because changes in systemic fluid balance can translate into retinal thickness changes when the blood–retinal barrier is already weak [125].

### 4.7. Lipid Therapy with Retinal Benefits: Fenofibrate as a Systemic Retina-Protective Model

Fenofibrate is a key example showing that systemic therapy, beyond lowering glucose, can modify diabetic retinopathy outcomes. In ACCORD Eye, adding fenofibrate to statin therapy reduced diabetic retinopathy progression compared with statin therapy alone [81,126]. The Fenofibrate Intervention and Event Lowering in Diabetes (FIELD) study also reported reduced need for laser treatment for diabetic retinopathy with fenofibrate, and the interpretation in that trial suggested that the benefit was not fully explained by lipid level changes alone [127].

Mechanistically, fenofibrate activates PPARα signaling and can change lipid handling, but it can also change inflammatory gene programs and endothelial function signals in ways that could support microvascular stability [81,126]. In the retina, a plausible model is that reduced inflammatory activation in endothelial cells and glial cells lowers leakage pressure over time, and that improved vascular stability reduces the pace of nonperfusion progression. Taken together, ACCORD Eye and FIELD show that diabetic retinopathy progression is shaped by the full vascular–metabolic environment, not only by ocular injections or glucose targets.

## 5. Perioperative Medication Issues Relevant to Systemic and Retinal Stress

A practical way to interpret perioperative retinal vulnerability is to consider four domains together rather than in isolation: baseline retinal status, baseline glycemic context and expected glycemic change, the type and stress burden of surgery, and perioperative medication interruption or continuation. These domains do not create a validated clinical algorithm, but they provide a structured framework for identifying settings in which short-term retinal instability may be more likely (Table 2).

### SGLT2 Inhibitors, Perioperative Ketoacidosis Risk, and Possible Relevance to Retinal Vulnerability

SGLT2 inhibitors increase ketone production risk during physiological stress because they shift fuel use toward fat oxidation and raise glucagon-to-insulin balance. Surgery adds fasting, catecholamines, cortisol, and relative insulin deficiency, so ketogenesis can rise even when glucose is not very high. This is why perioperative euglycemic DKA is a specific concern with this drug class [128,129].

Current U.S. regulatory guidance recommends preoperative interruption of SGLT2 inhibitors before scheduled surgery, generally 3 days for most agents and 4 days for ertugliflozin. These recommendations are relevant to the present review because perioperative ketoacidosis, dehydration, and hemodynamic instability may increase systemic stress and could, in theory, aggravate retinal vulnerability [130]. However, direct retina-specific evidence in this setting remains limited.

Restart should be conservative because the risk is driven by the postoperative metabolic state, not by the calendar alone. A practical restart standard is to resume only when the patient is clinically stable and hemodynamically stable, when oral intake is reliable, and when there is no evidence of ketosis or ongoing catabolic stress. If there are symptoms such as nausea, vomiting, abdominal pain, tachypnea, or unexplained anion-gap acidosis, clinicians should check ketones and acid–base status before considering restart. This approach reduces the chance that a patient leaves the hospital in a ketotic state while glucose looks acceptable [131].

**Figure 3:**
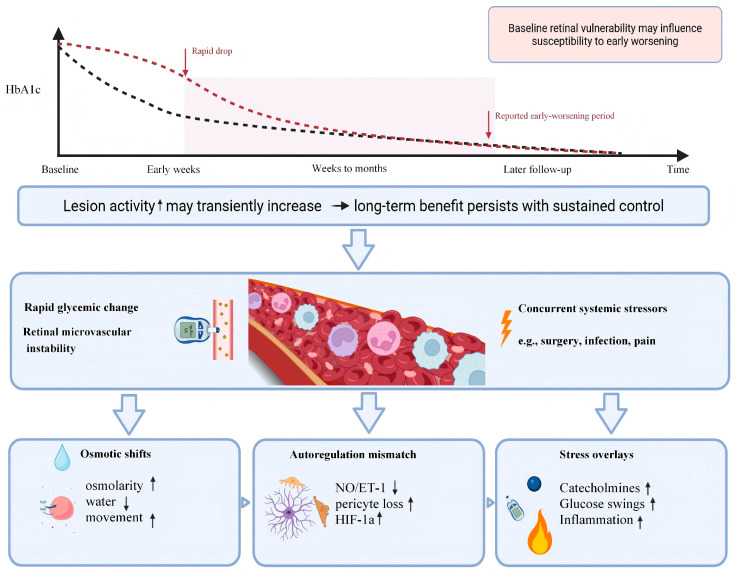
Proposed interactions among rapid glycemic change, systemic stress, and transient retinal vulnerability in diabetes. This figure is intended as a conceptual schematic rather than a clinical pathway. It summarizes how rapid glycemic change and concurrent systemic stressors may overlap with a period of transient retinal vulnerability in diabetes. The diagram highlights three proposed mechanism groups—osmotic shifts, autoregulatory mismatch, and systemic stress/inflammatory signaling—and their possible links to vascular leak, edema, hypoxia-related signaling, and transient lesion worsening. Baseline retinal vulnerability may modify susceptibility, whereas long-term retinal benefit may still persist with sustained glycemic control.

This is indirectly relevant to retinal risk management because severe postoperative metabolic decompensation drives systemic inflammation, dehydration, and perfusion instability, and these changes can worsen microvascular injury in many organs, including the retina. The practical point for a retina-focused review is simple: safe perioperative stopping and careful restart of SGLT2 inhibitors reduces the chance of a systemic crisis that can destabilize microvascular beds during recovery [128,129,130,131] (Table 3) (Figure 3).

## 6. Ophthalmic Surgery

### 6.1. Baseline Retinal Status and Key Surgical Considerations in Ophthalmic Surgery

Because ophthalmic surgery occurs in eyes that may already carry diabetic microvascular injury, baseline retinal phenotype is relevant when interpreting perioperative retinal outcomes [132,133]. Within the broader perioperative framework, ophthalmic surgery represents the setting in which baseline retinal status is likely to carry the greatest local relevance. In moderate-to-severe nonproliferative DR and proliferative DR, capillary nonperfusion is common, VEGF signaling is often high, and the blood–retinal barrier is fragile [6,132,133]. In this setting, surgical inflammation can push vascular leakage and edema. Cataract surgery is a typical example because it can increase postoperative inflammatory mediators in the anterior segment and the retina, and this can weaken barrier control and increase fluid movement into the macula [134,135]. Reviews focused on diabetic macular edema (DME) around cataract surgery describe a consistent mechanism: surgery-related inflammation can disrupt the blood–retinal barrier, so macular edema can worsen or appear after surgery in susceptible eyes [136].

This mechanism can be described at the cell level. Retinal endothelial cells rely on tight-junction proteins to limit leakage, and diabetes reduces junction stability through oxidative stress and inflammatory signaling [132,133]. Müller glia regulate water movement through aquaporin channels and support ionic balance through potassium channels, and diabetes shifts Müller cells toward a stress phenotype that is less stable for fluid control [6,133]. Microglia can move toward an activated state and release cytokines such as IL-1β and TNF-α, and these signals can further weaken endothelial junction function and increase leakage [6]. When cataract surgery adds an acute inflammatory pulse, these same cell groups are the ones that translate systemic and local inflammation into edema risk [135,136]. This helps explain why baseline retinal phenotype may influence the degree of perioperative edema risk and postoperative retinal response [133,135].

In the literature, pre-existing proliferative disease or center-involving DME is often discussed as a context that may complicate postoperative recovery and interpretation of surgical outcomes. Proliferative DR is driven by VEGF and ischemia, so neovascular tissue is fragile and prone to bleeding [133]. Center-involving DME is driven by leakage and inflammation, so it can worsen after surgery and reduce visual recovery even if the cataract operation is technically successful. OCT is helpful because it gives an objective baseline for macular thickness and fluid compartments, and it helps separate cataract-related blur from retinal disease after surgery [133,135]. OCTA can add information on nonperfusion burden in selected cases. Greater ischemic burden may also help explain why some eyes show stronger VEGF-related activity and higher recurrence risk, although VEGF-related signaling is not a routine clinical imaging endpoint [6,133].

For vitrectomy in proliferative DR, preoperative anti-VEGF can reduce intraoperative bleeding and can make surgery easier because neovascularization regresses. Timing matters because very long delays can allow fibrovascular tissue changes that increase traction risk in some eyes, while a short interval can capture the benefit of regression [137]. A large cohort analysis reported that a 4–7-day injection-to-surgery interval was linked with the lowest postoperative recurrence rates, while delays beyond 14 days were linked with higher tractional risk signals. Retina practice reviews often describe a similar window of roughly 3–7 days to allow regression while limiting traction concerns [138]. This is not a universal rule for every eye, but it explains why “anti-VEGF before vitrectomy” should be treated as a timing decision, not only a yes/no decision [137,138].

Retinal laser and intravitreal injections also interact with perioperative physiology [136]. Laser induces a controlled injury response, and injections can transiently change intraocular pressure and local inflammation [135]. These effects are usually small compared with the underlying disease, but they can matter when glucose is unstable and inflammation is high [132]. Perioperative glucose variability is relevant because hyperglycemia increases oxidative stress in endothelial cells and raises inflammatory tone in immune cells, and hypoglycemia episodes can trigger counter-regulatory hormones that increase variability again [6,132]. In eye surgery patients, a reasonable goal is stable perioperative glucose control in the recommended hospital range, not aggressive “tight control” that increases hypoglycemia risk. The American diabetes association (ADA) hospital standards recommend a perioperative glucose range of about 100–180 mg/dL for most patients [132].

### 6.2. Non-Ophthalmic Surgery: Retinal Risk from Systemic Stress and Treatment Disruption

By contrast, non-ophthalmic surgery does not directly injure retinal tissue, but it may still increase retinal vulnerability through systemic stress, hemodynamic change, inflammation, and medication disruption. Surgery increases catecholamines and cortisol, and it increases inflammatory mediators, and it can shift volume status and oxygen delivery. In diabetes, baseline endothelial function is often impaired, and inflammatory signaling is often higher, so the same stress response can cause a larger microvascular impact. Reviews of microvascular complications describe how hyperglycemia and insulin resistance create a proinflammatory environment through AGE–RAGE signaling, reactive oxygen species, and hypoxia-related pathways, and these pathways contribute to retinopathy and nephropathy in parallel [31,67,139]. Experimental work in diabetic microcirculation also supports that inflammatory cytokines such as IL-6 and TNF-α can drive endothelial dysfunction through oxidative stress and reduced endothelial nitric oxide synthase signaling, which is a relevant mechanism when surgery raises inflammatory load [139,140].

From a retinal viewpoint, several perioperative factors are high-yield. Hypotension and anemia reduce oxygen delivery, and this can amplify retinal hypoxia signals in eyes with existing nonperfusion. Dehydration and large fluid shifts change viscosity and perfusion pressure, and this can worsen capillary flow instability. Infection and severe pain increase cytokines and stress hormones, and this can increase VEGF pressure indirectly through hypoxia and inflammation [31,140]. Medication disruption is also common. SGLT2 inhibitors are stopped before surgery because of euglycemic DKA risk, and GLP-1RA plans may change due to aspiration concerns, and oral intake is delayed after some operations. These changes can increase glucose variability, and they can trigger short periods of severe hyperglycemia, and this can increase oxidative stress in endothelial cells and glia [31,139,141].

This is why high-risk eyes may need proactive follow-up after major systemic stress, even when the surgery is far from the eye. “High risk” here means severe NPDR or proliferative DR, or center-involving DME, or a recent large HbA1c drop with therapy intensification, or combined nephropathy and hypertension that signals broad microvascular fragility [31,67]. These patients may represent a setting in which early retinal changes deserve careful interpretation, especially when systemic stress overlaps with rapid glycemic change. However, the available evidence remains indirect and does not define a formal post-stressor surveillance protocol.

Taken together, perioperative retinal vulnerability is likely to be highest when four features overlap: advanced baseline DR, poor baseline glycemic control or recent rapid HbA1c reduction, a higher-stress surgical setting, and major medication or insulin disruption. By contrast, risk is likely lower when baseline DR is absent or mild, glycemic change is modest, surgery is less inflammatory, and perioperative metabolic control remains stable. This framework is intended to organize current evidence rather than to function as a validated clinical algorithm.

## 7. Gaps, Controversies, and Research Priorities

The main controversy is whether observed diabetic retinopathy signals with SGLT2 inhibitors and GLP-1RAs reflect true drug effects or baseline differences between treated groups [142]. Real-world comparisons often mix patients with different HbA1c, diabetes duration, kidney disease, blood pressure, baseline retinopathy stage, and eye-care intensity, so confounding can easily create “benefit” or “harm” patterns [143]. A priority is better causal designs, including new-user active-comparator cohorts with documented baseline DR severity and imaging, strong adjustment for eye-care utilization [124], and analyses that separate early EWDR-like periods from longer follow-up where chronic vascular remodeling dominates [144].

A second gap is that most metabolic trials lack retina-centered clinical endpoints. Future trials could incorporate standardized OCT and optical coherence tomography angiography (OCTA) endpoints to quantify macular edema, capillary nonperfusion, and microvascular density. Mechanistic correlates of NVU stress, including VEGF-related signaling, endothelial barrier injury, and inflammatory pathways involving microglia and Müller glia, may be better addressed in translational substudies or experimental systems rather than as routine patient-level trial endpoints. In parallel, research should define a “safe velocity” of HbA1c reduction for high-risk eyes by modeling HbA1c slope, Continuous glucose monitoring variability metrics, and perioperative hemodynamics as time-linked exposures, then testing how these exposures predict short-term EWDR and longer-term DR progression [65].

## 8. Conclusions

Modern antihyperglycemic therapies improve glucose control and also improve blood pressure, weight, and kidney and cardiovascular risk in many patients, so they can support long-term retinal protection through better systemic vascular health [142,143]. The key short-term issue is EWDR, which can occur when HbA1c falls quickly in patients with moderate-to-severe baseline DR, long-standing poor control, or coexisting nephropathy and hypertension [145]. The semaglutide signal in SUSTAIN-6 matches this EWDR-like pattern because events were mostly early and were concentrated in patients with pre-existing DR and large HbA1c reductions [142,143]. Taken together, current evidence suggests that baseline retinal status, the magnitude and speed of HbA1c reduction, and the presence of perioperative stress may all influence short-term retinal vulnerability. These observations support careful interpretation of early retinal events after treatment intensification, rather than a drug-class-specific toxic explanation.

Perioperative care adds metabolic stress, hemodynamic shifts, inflammation, and drug interruptions, and these factors can increase glucose variability and microvascular vulnerability during recovery. Most inpatient standards target perioperative glucose around 100–180 mg/dL to limit infection risk while reducing hypoglycemia, and SGLT2 inhibitors should be stopped before scheduled surgery to reduce euglycemic DKA risk, with restart only after stable oral intake and low ketosis risk [113,146]. GLP-1RA guidance has moved toward risk-based continuation for most elective cases, with individualized holding plans for patients with high aspiration risk or significant GI symptoms. Overall, the literature suggests that retinal outcomes in diabetes may be shaped by the interaction of chronic metabolic control, rapid glycemic change, and perioperative systemic stress [113,142,143,145,146]. Because much of the available evidence is indirect, heterogeneous, or mechanistic, these links should be viewed as evidence-informed considerations rather than formal practice recommendations.

## Figures and Tables

**Table 1:** Clinical factors associated with reported EWDR risk in the literature.

Risk Tier	Baseline Retinal Status (Most Recent Exam)	Baseline Glycemia and Expected “A1c Velocity”	Typical Trigger Situations	Why This Setting May Be Associated with EWDR Risk	Timing of Reported Early Worsening in the Literature	Clinical Interpretation and Remaining Uncertainty
Low	No DR or mild NPDR; no prior Diabetic macular edema (DME)	HbA1c < 9% or expected drop < 1% over ~3 months	Starting GLP-1RA/SGLT2i/metformin without major insulin changes	Reported EWDR risk appears low when baseline DR is absent or mild and expected HbA1c change is small.	No distinct early-worsening window has been consistently described in this setting	This category is included mainly as a reference group; the direct evidence remains limited
Moderate	Mild–moderate NPDR or remote history of DME now stable	HbA1c 9–11% or expected drop ~1–2% in 3 months	Dose escalation of GLP-1RA; adding basal insulin; switching to higher potency therapy	Moderate baseline retinal disease plus a larger expected HbA1c decline may increase short-term retinal vulnerability	When reported, early worsening has generally been described within weeks to a few months after intensification	Baseline retinal phenotype may help contextualize risk, but current evidence is insufficient to define a standard surveillance schedule
High	Severe NPDR or PDR, active or recently treated; and/or center-involving DME	HbA1c ≥ 11% or expected drop ≥ 2% within ~8–12 weeks	Rapid A1c improvement after starting potent GLP-1RA/dual incretin, intensive insulin titration, or multiple agent add-on	This setting combines advanced retinal disease with rapid metabolic change, which is the pattern most often associated with reported EWDR	Reported early worsening is most often described in the first several weeks to months after intensification	Available evidence supports heightened interpretive caution in this group, but not a uniform evidence-based clinical algorithm
Very high/fragile	PDR with recent VH/traction risk; uncontrolled DME with major VA loss; extensive ischemia	HbA1c very high (often ≥12–13%) with planned aggressive correction	“Rescue” glycemic correction (high-dose insulin, inpatient transitions), major systemic stress (infection/surgery) during escalation	Overlapping retinal, metabolic, and systemic stressors may plausibly amplify short-term retinal instability	The literature is sparse and largely indirect, so the timing and magnitude of risk remain uncertain	This category should be viewed as a hypothesis-generating summary rather than a validated management tier

**Table 2:** Structured perioperative retinal-vulnerability framework.

Domain	Lower-Concern Setting	Higher-Concern Setting	Why It May Matter
Baseline retinal status	No DR or mild NPDR	Severe NPDR, proliferative diabetic retinopathy, or center-involving DME	Lower retinal reserve and greater leak/ischemic burden
Glycemic context	Modest HbA1c change, no recent treatment intensification	High baseline HbA1c, recent rapid HbA1c reduction, or recent intensification	Greater overlap with an EWDR-prone period
Surgical context	Minor low-stress procedure	Ophthalmic surgery in a diseased eye or major non-ophthalmic surgery with inflammatory/hemodynamic stress	Greater inflammation, fluid shift, and perfusion instability
Medication context	Minimal interruption, stable oral intake	Major insulin disruption, SGLT2i interruption with ketosis risk, delayed intake, or marked perioperative variability	Greater glucose instability and systemic metabolic stress

**Table 3:** Perioperative medication issues discussed in current guidelines and their possible relevance to retinal vulnerability.

Medication Class	Examples	Perioperative Issue Described in Current Guidelines and Reviews	Main Perioperative Concern	Relevant Perioperative Context	Conditions for Medication Resumption Described in Current Guidance	Possible Relevance to Retinal Vulnerability	Evidence Basis/Retina-Specific Limitation
SGLT2 inhibitors	empagliflozin, dapagliflozin, canagliflozin, ertugliflozin	Current guidance generally recommends preoperative interruption, usually 3 days before surgery for most agents and 4 days for ertugliflozin	Euglycemic DKA risk with fasting + stress + relative insulin deficiency	Perioperative reviews describe glucose and ketone assessment as relevant when ketoacidosis risk is suspected or clinically increased	Resumption is generally described after recovery of hemodynamic stability, oral intake, and resolution of ketosis concern	These measures may reduce systemic stressors that could indirectly aggravate retinal vulnerability, although direct retina-specific evidence remains limited	Regulatory and perioperative guideline based for systemic safety; retinal relevance is indirect and supported mainly by mechanistic inference
GLP-1 receptor agonists/dual incretins	semaglutide, liraglutide, dulaglutide; tirzepatide (dual)	Current multisociety guidance generally supports continuation in most patients, while selected higher-aspiration-risk settings are discussed separately	Delayed gastric emptying → aspiration risk; risk higher during dose escalation, higher doses, and with GI symptoms	Published guidance focuses on aspiration-related context, especially during dose escalation or in the presence of gastrointestinal symptoms	Continuation or resumption is generally described relative to postoperative intake and aspiration-risk context rather than retina-specific evidence	Any retinal relevance is likely indirect and mediated through overall glycemic stability rather than a direct ocular effect	Guideline and multisociety consensus based for aspiration-risk management; retina-specific evidence is indirect and mainly extrapolated from EWDR-related glycemic change literature
Basal insulin (glargine/detemir/degludec/Neutral protamine Hagedorn (NPH) basal component)	long-acting basal; NPH	Usually continue, often at a reduced dose (institution-specific; commonly modest reduction morning-of)	Complete basal withdrawal → ketosis risk (esp type 1 diabetes, insulin-deficient type 2 diabetes)	Use protocolized basal + correction; for major surgery/critical illness consider IV insulin protocol with frequent checks	Continue uninterrupted; titrate as stress resolves and diet resumes	Stable basal coverage reduces glucose swings (variability) that can stress endothelial junctions and worsen edema tendency	Perioperative diabetes management guidance based; retinal relevance is indirect and linked to avoidance of major glycemic variability rather than direct retinal trials
Prandial insulin	lispro/aspart/glulisine	Hold while Nil per os (nothing by mouth); resume with meals	Hypoglycemia if given without intake	Use correctional insulin with scheduled monitoring; restart prandial dosing with first meal and adjust for intake	Restart when eating; match to carbs/meal size	Prevent large hyperglycemia spikes post-op (which increase ROS/inflammatory signaling) and avoid rebound variability from hypoglycemia rescue	General perioperative glycemic management guidance; no direct retina-specific perioperative trial evidence
Insulin pump/hybrid closed-loop	Continuous subcutaneous insulin infusion/Automated insulin delivery systems	Depends on surgery length/setting; many centers allow continuation for short procedures with anesthesia agreement	Device displacement, altered absorption, safety oversight	Have a clear institutional plan: who manages pump, target range, backup basal plan if stopped	Restart per protocol with verified settings and stable intake	Maintains smoother glycemia and reduces variability if safely managed	Institutional perioperative diabetes protocols and anesthesia/device safety considerations; Indirect relevance through glycemic stability; no direct retina-specific perioperative trial evidence
Metformin	metformin	Often hold day-of and during hemodynamic instability/Acute kidney injury (AKI) risk (institution-specific)	Lactic acidosis risk in renal dysfunction/hypoperfusion	Manage glucose with insulin strategy while held	Restart when renal function/perfusion stable and eating	Avoid uncontrolled hyperglycemia during the hold; hyperglycemia + dehydration can worsen microvascular stress	General perioperative safety guidance related to renal dysfunction, hypoperfusion, and lactic acidosis risk; Indirect relevance through avoidance of severe hyperglycemia and dehydration; no direct retina-specific perioperative evidence
Sulfonylureas	glipizide, glyburide	Usually hold day-of (often earlier for long-acting agents)	Hypoglycemia risk with fasting	Prefer insulin-based inpatient control if needed	Restart when regular diet and low hypoglycemia risk	Hypoglycemia → counter-regulatory surge → rebound hyperglycemia/variability (bad for NVU stability)	Standard fasting-related hypoglycemia prevention principles; Indirect relevance through glycemic variability and counter-regulatory stress; no drug-specific retinal perioperative signal
DPP-4 inhibitors	sitagliptin, linagliptin	Variable; some centers continue, others stop with other orals	Generally low hypoglycemia risk	If continued, still monitor; if stopped, ensure insulin coverage	Restart when diet stable	Low potency—retinal relevance is mostly via overall glycemic stability, not drug-specific eye signals	Local perioperative practice patterns and low hypoglycemia risk profile; Limited and indirect retinal relevance; no strong evidence for a specific perioperative retinal effect
TZDs	pioglitazone	Often avoided periop if fluid risk; not a standard acute inpatient tool	Fluid retention; Congestive heart failure risk	If held, compensate with insulin plan	Restart only if fluid status stable and benefits outweigh edema risk	Fluid shifts can worsen DME susceptibility in vulnerable eyes—avoid piling on edema drivers post-op	Supported mainly by case reports and safety discussions; population-level retinal evidence remains inconsistent

## Data Availability

No new data were created or analyzed in this study.

## Abbreviations

The following abbreviations are used in this manuscript:

ACCORD	Action to Control Cardiovascular Risk in Diabetes
ADA	American diabetes association
AGE(s)	Advanced glycation end product(s)
AGE-RAGE	Advanced glycation end products–receptor for advanced glycation end products
AID	Automated insulin delivery
AKI	Acute kidney injury
AMPK	AMP-activated protein kinase
anti-VEGF	Anti-vascular endothelial growth factor
cAMP	Cyclic adenosine monophosphate
CCL2	C-C motif chemokine ligand 2
CD68	Cluster of differentiation 68
DKA	Diabetic ketoacidosis
DME	Diabetic macular edema
DNA	Deoxyribonucleic acid
DPP-4	Dipeptidyl peptidase-4
DR	Diabetic retinopathy
EAAT1	Excitatory amino acid transporter 1
EWDR	Early worsening of diabetic retinopathy
FIELD	Fenofibrate Intervention and Event Lowering in Diabetes
GFAP	Glial fibrillary acidic protein
GLP-1	Glucagon-like peptide-1
GLP-1RA	Glucagon-like peptide-1 receptor agonist
HbA1c	Glycated hemoglobin
HIF-1α	Hypoxia-inducible factor-1 alpha
ICAM-1	Intercellular adhesion molecule-1
IL-1β	Interleukin-1 beta
IL-6	Interleukin-6
IV	Intravenous
Iba1	Ionized calcium-binding adaptor molecule 1
Kir4.1	Inwardly rectifying potassium channel 4.1
NADPH	Nicotinamide adenine dinucleotide phosphate
NF-κB	Nuclear factor kappa B
NLRP3	NLR family pyrin domain containing 3
NPDR	Non-proliferative diabetic retinopathy
NPH	Neutral protamine Hagedorn
NVU	Neurovascular unit
OCT	Optical coherence tomography
OCTA	Optical coherence tomography angiography
PDGF	Platelet-derived growth factor
PDGF-B	Platelet-derived growth factor B
PDGFR-β	Platelet-derived growth factor receptor beta
PKA	Protein kinase A
PKC	Protein kinase C
PKC-β	Protein kinase C beta
PPARα	Peroxisome proliferator-activated receptor alpha
PPARγ	Peroxisome proliferator-activated receptor gamma
PRP	Panretinal photocoagulation
RAGE	Receptor for advanced glycation end products
ROS	Reactive oxygen species
SGLT2	Sodium-glucose cotransporter 2
SGLT2i	Sodium-glucose cotransporter 2 inhibitor
SUSTAIN-6	Semaglutide Unabated Sustainability in Treatment of Type 2 Diabetes 6
Tie2	Tyrosine kinase with immunoglobulin and EGF homology domains 2
TNF-α	Tumor necrosis factor alpha
TZD	Thiazolidinedione
VA	Visual acuity
VCAM-1	Vascular cell adhesion molecule-1
VEGF	Vascular endothelial growth factor
VH	Vitreous hemorrhage
